# Comparative antibacterial activity of N-terminal and C-terminal domains of a recombinant endolysin against *Cutibacterium acnes*

**DOI:** 10.1128/aem.01168-25

**Published:** 2025-09-22

**Authors:** Jae-Hyuk Lee, Muhammad Adeel Hasnain, Jung-Ho Park, Wonho Choi, Gi-Seong Moon

**Affiliations:** 1 Department of Biotechnology, Korea National University of Transportation, Jeungpyeong, Republic of Korea; 2 Major in IT·Biohealth Convergence, Department of IT·Energy Convergence, Graduate School, Korea National University of Transportation, Chungju, Republic of Korea; 3 Bio-Evaluation Center, Korea Research Institute of Bioscience and Biotechnology 54679, Cheongju, Republic of Korea; 4 Department of Applied Biological Engineering, KRIBB School of Biotechnology, Korea University of Science and Technology (UST), Daejeon, Republic of Korea; 5 4D Convergence Technology Institute, Korea National University of Transportation, Jeungpyeong, Republic of Korea; 6 Future Innovation Materials Global Leading Research Center (Regional Leading Research Center), Korea National University of Transportation, Jeungpyeong, Republic of Korea; University of Nebraska-Lincoln, Lincoln, Nebraska, USA

**Keywords:** endolysin, host specificity, antibiotic resistance, *Cutibacterium acnes*, protein-based therapeutics

## Abstract

Endolysins are promising antibacterial agents due to their selective antibacterial activity and minimal risk of resistance. The antibacterial activity of individual domains of endolysins from anti-*Cutibacterium acnes* phages remains unexplored. In this study, a recombinant endolysin from *C. acnes* phage CAP 10-3 and its N-terminal and C-terminal domains were tested against *C. acnes* KCTC 3314 and KCTC 3320 using turbidity reduction and live cell count methods. Starting with an initial OD_600_**≈**1, a 3 h treatment with 50 µg/mL of the N-terminal domain (N1) reduced OD_600_ to 0.395 (KCTC 3314) and 0.324 (KCTC 3320), while that of full-length (FL) reduced OD_600_ to 0.547 and 0.474, respectively. Correspondingly, N1 reduced viable counts by 1.13–1.35 log, whereas FL showed 0.94–0.95 log reduction, confirming N1’s stronger bactericidal effect. C-terminal domain exhibited minimal changes in both OD_600_ and cell count values. N1’s lytic effect was further confirmed by morphology analysis. Therefore, the N-terminal domain was further characterized for its activity and specificity. It showed dose-dependent activity against both *C. acnes* strains, but it had no significant effect on *Staphylococcus aureus* KCTC 3881 and only weak activity against *Staphylococcus epidermidis* CJNU 0702 at high concentrations (100 µg/mL). These findings highlight the N-terminal domain’s potential as a selective anti-*C*. *acnes* agent with minimal impact on skin flora, supporting its suitability for protein-based antimicrobial therapeutics and cosmetic applications.

**IMPORTANCE:**

*Cutibacterium acnes* is known to play a significant role in the pathology of acne vulgaris and several other disorders. Conventional methods of treating *C. acnes* infections using antibiotics face an ever-aggravating antibiotic resistance challenge. Endolysins present a promising alternative with advantages such as specificity and low-to-no chances of resistance. The current study compares the antibacterial activity of the full length as well as its N- and C-terminal domains of an endolysin from phage CAP 10-3. In addition, the dose-dependent effect and specificity of the N-terminal domain (which showed the most significant anti-*C*. *acnes* activity) are also explained. These findings can pave the way for developing alternative peptide-based anti-*C*. *acnes* therapeutics.

## INTRODUCTION

Antibiotics represent a groundbreaking medical discovery for the treatment of infectious diseases. However, prolonged overuse and misuse of antibiotics have led to a rapid increase in bacterial resistance to antibiotics (1, 2). This resistance significantly reduces the efficacy of conventional therapies, thereby necessitating the search for novel alternative treatment strategies (3–5). Among these alternatives, bacteriophage-derived endolysins have emerged as a promising antimicrobial agent with potent bactericidal activity, offering an innovative therapeutic solution that overcomes the limitations of traditional antibiotics.

Endolysins, essential enzymes in the bacteriophage life cycle, degrade the peptidoglycan (PG) layer of the bacterial cell wall, facilitating phage progeny release (6). Endolysins from the phages infecting Gram-positive bacteria are usually composed of two types of functional domains, that is, the enzymatically active domain (EAD) and a cell wall binding domain (CBD) (7). EAD of endolysins includes amidases, glycosidases, endopeptidases, and muramidases, which target glycosidic, amide, or peptide bonds in PG (8, 9). The CBD provides substrate specificity by selectively binding to bacterial PG (10, 11). The structural characteristics of endolysins vary, with their domains arranged in a non-uniform manner, regardless of the N-terminal or C-terminal orientation. In some cases, endolysins may lack a distinct CBD altogether (12, 13). Endolysins hold significant potential for overcoming the limitations of conventional antibiotics as powerful tools capable of selectively infecting and eliminating specific bacteria. Use of endolysins has garnered more attraction than that of whole phages since phage therapy is limited by bacterial host receptor mutations, which can alter phage binding sites, and bacterial defense mechanisms such as CRISPR-Cas systems, which can cleave phage genetic material. Additional concerns include a lack of pharmacokinetic data, the potential release of endotoxins due to excessive bacterial lysis, and allergic reactions (14–16).

*Cutibacterium acnes* (formerly *Propionibacterium acnes*) is a Gram-positive, aerotolerant, anaerobic bacterium frequently isolated from the sebaceous glands of the skin (17). *C. acnes* is a key opportunistic pathogen implicated in acne and various other inflammatory skin disorders (18, 19). Excessive proliferation of *C. acnes* activates the innate immune system, particularly through the Toll-like receptor 2 pathway, leading to the production of pro-inflammatory cytokines, including IL-1β, IL-8, TNF-α, and IFN-γ (20). This inflammatory cascade is further exacerbated by metabolic byproducts of *C. acnes*, such as short-chain fatty acids and porphyrins, which contribute to follicular damage and inflammation (21–23).

Current therapeutic approaches for mitigating *C. acnes* primarily involve administration of topical and systemic antibiotics such as clindamycin and tetracycline. However, the overuse of these antibiotics has resulted in an alarming rise in *C. acnes* antibiotic resistance globally (4, 24, 25). This resistance not only compromises skin homeostasis but also emphasizes the urgent need for alternative therapeutic strategies (26). Controlling *C. acnes* proliferation remains a critical challenge in the prevention of acne and related inflammatory disorders.

To date, only a few studies have analyzed the antibacterial potential of endolysins derived from anti-*C*. *acnes* phages (27, 28), while the antibacterial activity of the individual domains (C-terminal and N-terminal) is yet to be evaluated. Challenges such as low protein yield during purification and uncertainties regarding their effect on commensal skin bacteria remain unresolved. Endolysins derived from *C. acnes* phages are predicted to possess an N-terminal EAD and a C-terminal domain with unknown function (29). Therefore, the current study focused on enhanced expression of CAP 10-3 endolysin in soluble, active form using a pET-28α-based protein expression system in *Escherichia coli* BL21(DE3). The antibacterial activity of the full-length (FL) protein as well as its N-terminal and C-terminal domains was evaluated against two *C. acnes* strains. Additionally, specificity of N-terminal domain was also analyzed by assessing its antibacterial activity against the common skin inhabitants, that is., *Staphylococcus aureus* and *Staphylococcus epidermidis*.

## MATERIALS AND METHODS

### Bacterial strains and culture conditions

The bacterial strains used in this study included *C. acnes* KCTC 3314 (isolated from acne lesions on human facial skin), *C. acnes* KCTC 3320 (isolated from a human subcutaneous abscess), and *S. aureus* KCTC 3881 which were purchased from the Korean Collection for Type Cultures (KCTC; Jeongeup, Korea). *S. epidermidis* CJNU 0702 was isolated from human breast milk. *E. coli* DH5α (used for cloning), and *E. coli* BL21 (DE3) (used for the protein expression) were purchased from Ezynomics (Ezynomics, Inc., Daejeon, Korea). The cultivation of *C. acnes* KCTC 3314 and KCTC 3320 was carried out using Reinforced Clostridial Medium (RCM) broth (BD, Sparks, MD, USA). For the assessment of antimicrobial activity based on turbidity analysis and viable cell counts (Log CFU/mL), the RCM broth was centrifuged (3,600 × *g*, 4°C, 10 min), and the agar content was removed before sterilization. The cultures of *C. acnes* KCTC 3314 and KCTC 3320 were maintained under anaerobic conditions using an anaerobic chamber (DG250; Don Whitley Scientific Ltd., Bingley, UK). *S. aureus* KCTC 3881 and *S. epidermidis* CJNU 0702 were cultivated in MRS broth (BD). For *E. coli* strains DH5α and BL21(DE3), Luria-Bertani broth (BD) was used, and the cultures were incubated at 37°C with shaking at 200 rpm.

### Prediction of secondary structure of endolysin

The CAP10-3 endolysin sequence was extracted from GenBank accession no. OR039357. The secondary structure of N-terminal domain (1–185 aa), and C-terminal domain (181–285 aa) of the endolysin was predicted using AlphaFold (https://www.alphafold.ebi.ac.uk).

### Construction of recombinant plasmids

To obtain the FL, N-domain, and C-domain of CAP 10-3 derived endolysin, primers were designed with *NdeI* and *XhoI* restriction sites. The primer sequences for forward and reverse primers, respectively, were as follows: (i) FL: F-tatacatatgGTGAGGTTTATTCC, R-tataCTCGAGCTTCTTTAAACCGT; (ii) N-terminal domain: F-tatacatatgAGGTTTATTCCTGCA, R-tatactcgagGCAGACGACGGCCAT; and (iii) C-terminal domain: F-tatacatATGGCCGTCGTCTGCGG, R-tataCTCGAGCTTCTTTAAACCGT. Endolysin gene (FL) and its respective domains were amplified using PCR (PC-812; Astec, TN, USA). PCR running conditions were as follows: 95°C, 5 min for initial denaturation; 30 cycles of 95 °C-30 s, 57 °C-30 s, and 72 °C-1 min; 72°C, 5 min for final extension. The protein expression vector pET-28α (Novagen, MA, USA) was used for efficient protein purification as it contains His tags on both the N-terminal and C-terminal ends. The amplified PCR product and the plasmid vector were digested with the restriction enzymes *NdeI* and *XhoI* (Takara Bio Inc., Kusatsu, Japan) and ligated using T4 DNA ligase (Bioneer Inc., Daejeon, Korea). The recombinant plasmid was transformed into *E. coli* DH5α via heat shock at 42°C for 15 s. Transformants containing the pET-28α vector were selected on LB agar plates supplemented with kanamycin (50 µg/mL). Recombinant plasmids were extracted from the transformed *E. coli* DH5α using a plasmid prep kit (iNtRON Biotechnology, Seongnam, Korea). The presence of each respective endolysin domain in the transformants was confirmed via PCR and DNA gel electrophoresis analysis. Subsequently, the recombinant plasmids were transformed into the protein expression host *E. coli* BL21(DE3) under the same transformation conditions as for *E. coli* DH5α. The recombinant *E. coli* BL21(DE3) transformants were verified using the same methods as described above.

### Protein expression and purification

To express recombinant endolysin and its domains, *E. coli* BL21(DE3) transformed with pET-28α_CAP10-3 (FL), pET-28α_CAP10-3_N1 (N-domain), and pET-28α_CAP10-3_C1 (C-domain) were inoculated at 3% (vol/vol) into 900 mL of LB broth containing kanamycin (50 µg/mL). The cultures were incubated at 37°C with shaking at 250 rpm until an OD_600_ of 0.4–0.6. Protein expression was induced by adding 0.5 mM isopropyl-β-d-thiogalactopyranoside (IPTG; Bioneer Inc., Daejeon, Korea) followed by overnight incubation at 25°C with shaking at 120 rpm. After overnight induction, bacterial cells were harvested in pellet through centrifugation (5,000 *× g*, 4°C for 40 min). The pellet was resuspended in 75 mL of lysis buffer (1× phosphate buffered saline [PBS], 10 mM β-mercaptoethanol) supplemented with a protease inhibitor cocktail (Sigma-Aldrich, St. Louis, MO, USA). The suspension was subjected to cell lysis three to four times using a French press VS-4600P (Vision Scientific, Daejeon, Korea). The lysate was then centrifuged at 12,000 *× g*, 4°C for 1 h to obtain the supernatant, which was then filtered through a 0.45 µm syringe filter (Anylab, Seoul, Korea). The filtered lysate was loaded onto an open column (Bio-Rad, Hercules, CA, USA) containing 2 mL of Ni-NTA agarose slurry (QIAGEN, Hilden, Germany) for protein purification. The column was washed first with wash buffer (1× PBS, 5 mM β-mercaptoethanol) and then with the wash buffer containing 20 mM imidazole (1× PBS, 20 mM imidazole, 5 mM β-mercaptoethanol). Finally, recombinant endolysin or its respective domains was eluted using elution buffer (1× PBS, 250 mM imidazole, 2 mM β-mercaptoethanol). Elution samples were analyzed by SDS-PAGE and Western blot. The eluted recombinant proteins were subjected to dialysis using a dialysis bag (Thermo Fisher Scientific, Waltham, MA, USA) in 2 L of dialysis buffer (1× PBS). The buffer was replaced twice at 12 h intervals to remove imidazole. Protein concentration was determined through bicinchoninic acid (BCA) assay using Thermo Scientific Pierce BCA kit (Thermo Fisher Scientific, Rockford, IL, USA), following the manufacturer’s protocol.

### Evaluation of antibacterial activity of recombinant endolysins

The antibacterial activity of the recombinant endolysin and its respective domains against *C. acnes* KCTC 3314 and KCTC 3320 was evaluated using a turbidity reduction assay and viable cell count (Log CFU/mL) based on the method previously described (27). To prepare the bacterial samples, *C. acnes* KCTC 3314 and KCTC 3320 were grown to mid-log phase. The pellet from 1 mL of target bacterial cells was washed twice with 1× PBS (Biosesang, Yongin, Korea). The concentration of cells was then adjusted to OD_600_ ≈1.0 and treated with the cell lysates containing recombinant proteins at a concentration of 300 µg/mL. The treated samples were incubated anaerobically at 37°C, and turbidity reduction and viable cell count (Log CFU/mL) were measured at 0, 2, 4, 6, and 8 h. In the case of purified proteins, a concentration of 50 µg/mL was used, and turbidity reduction and viable cell count (Log CFU/mL) were measured at 0.5 h intervals up to 3 h. To further assess the dose-dependent antibacterial activity of pET-28α_CAP10-3_N1 (N-domain), *C. acnes* KCTC 3314 and KCTC 3320 were treated with N-domain at concentrations of 25, 50, and 100 µg/mL. Turbidity reduction and viable cell count were measured at 0.5 h intervals for up to 3 h.

### SEM imaging for morphology analysis

To observe the PG dissolution and cell morphology of *C. acnes* KCTC 3314 and KCTC 3320 following treatment with purified recombinant endolysin N-domain, scanning transmission electron microscopy (SEM EVO MA 10, Carl Zeiss, Jena, Germany) was utilized. For SEM imaging, *C. acnes* KCTC 3314 and KCTC 3320 in mid-log phase were treated with purified N-domain at a concentration of 100 µg/mL for 3 h. Following treatment, the bacterial cells were centrifuged at 21,000 *× g*, 4°C, for 3 min to remove the supernatant. The cell pellet was resuspended in an equal volume of 50% ethanol and incubated for 15 min. The samples were sequentially dehydrated with 60%, 70%, 80%, 95%, and 100% ethanol under the same conditions. The dehydrated samples were mounted onto an SEM-specific sample holder (Sigma-Aldrich) by attaching silicon wafer fragments (LG Siltron, Inc., Gumi, Korea) using SEM mounting adhesive (Sigma-Aldrich). The samples were then coated with platinum using an ion sputter coater (SPT-20, Coxem, Daejeon, Korea) at 5 mA for 120 s. To enhance conductivity, silver paint (Ted Pella, Inc., Redding, CA, USA) was applied around the sample. The morphology of *C. acnes* KCTC 3314 and KCTC 3320 cells was subsequently observed under SEM.

### Evaluation of the specificity of N-terminal domain

Since the N-terminal domain showed the highest antibacterial activity against both *C. acnes* strains, it was further evaluated for its specificity. Its antibacterial activity was assessed using a turbidity reduction assay and viable cell count (Log CFU/mL) against two common skin commensal bacteria, that is, *S. aureus* KCTC 3881 and *S. epidermidis* CJNU 0702. For this purpose, the target strains were grown to mid-log phase growth (6 h). The pellet of 1 mL of target bacterial cells was washed twice with 1× PBS. The concentration of bacterial cells was then adjusted to OD_600_ ≈1.0, and the cells were treated with various concentrations of the purified recombinant endolysin N1 (N-domain) and incubated at 37°C. Turbidity reduction and viable cell count (Log CFU/mL) were measured at 0.5-h intervals for up to 3 h. These measurements were compared to those for *C. acnes* KCTC 3314 and KCTC 3320 to confirm the specificity of the CAP10-3_N1 (N-domain)

### Statistical analysis

All turbidity reduction (OD_600_) and viable cell count (Log CFU/mL) experiments were performed in triplicate and expressed as mean ± standard deviation. Statistical analyzes were conducted using SPSS ver. 25 (Statistical Package for the Social Sciences, SPSS Inc., Chicago, IL, USA). One-way analysis of variance was used to evaluate statistical significance, and *post hoc* testing for absorbance (OD_600_) in the turbidity reduction assay and viable cell counts (Log CFU/mL) was conducted using Tukey’s honest significant difference test (*P* < 0.05).

## RESULTS

### Prediction of the secondary structure of CAP10-3 endolysin

**Fig 1:**
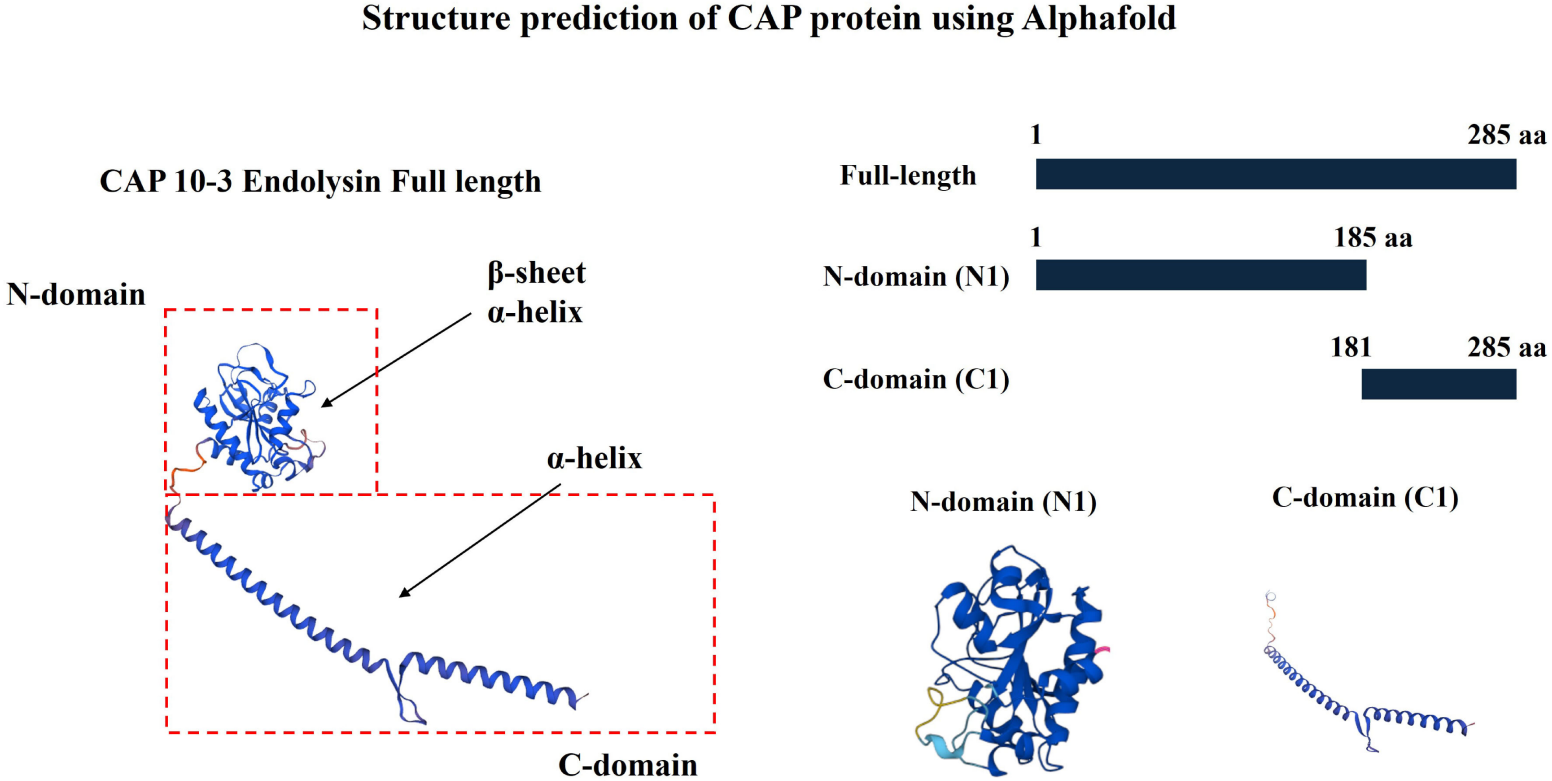
Predicted secondary structure of bacteriophage CAP10-3 endolysin using AlphaFold.

Using AlphaFold, the structural characteristics of bacteriophage CAP10-3 endolysin were predicted. The C-domain (181–285 aa) predominantly exhibited a linear structure composed of α-helices, while the N-domain (1-185 aa) displayed a combination of α-helices and β-sheets in its structure (Fig. 1).

### Expression and purification of recombinant endolysin

**Fig 2:**
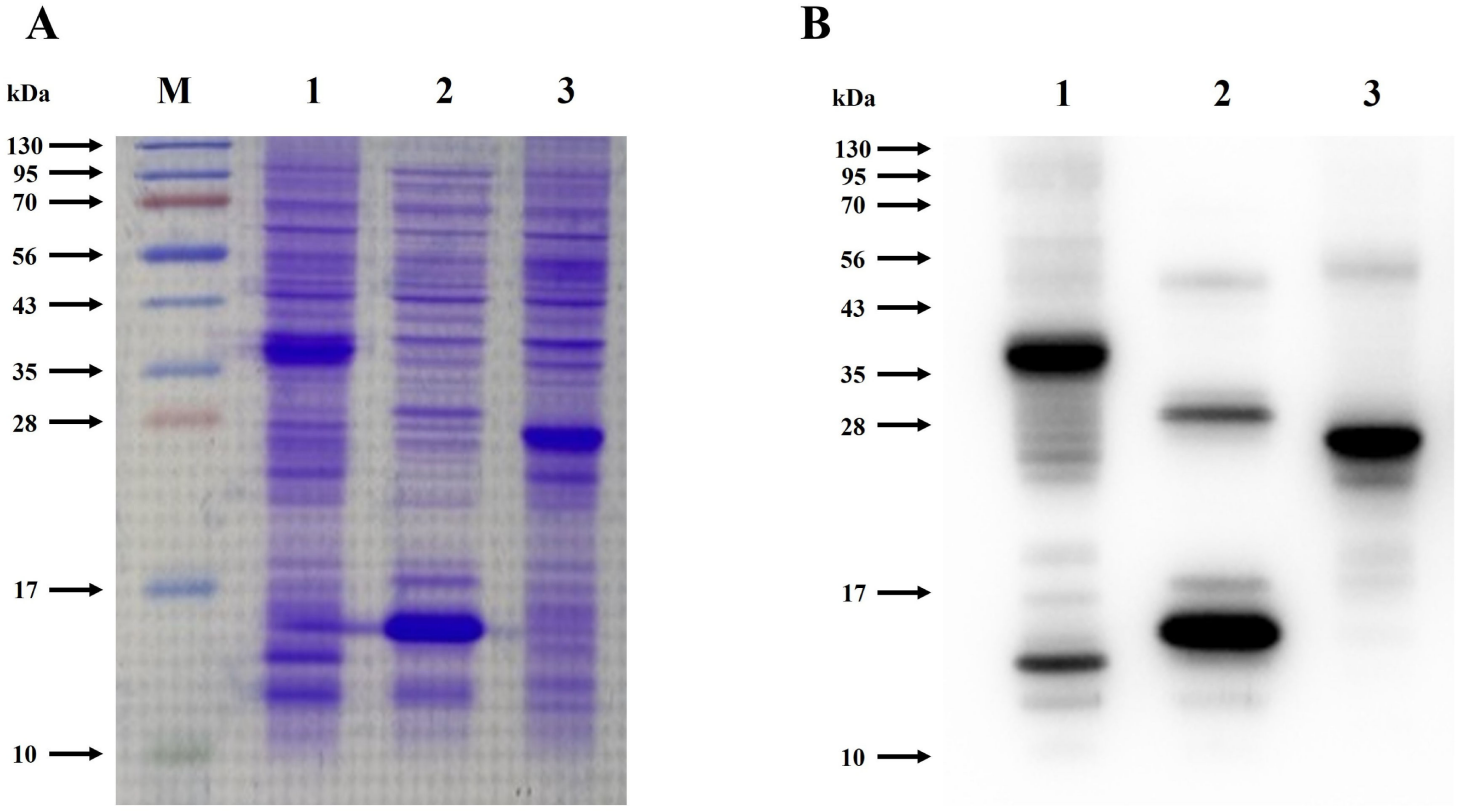
Expression of recombinant CAP10-3 endolysin and its domains in *E. coli* BL21(DE3). (**A**) SDS-PAGE analysis of total protein expression. (**B**) Western blot analysis of recombinant proteins. M: protein marker; Lane 1: FL; Lane 2: C-terminal domain; Lane 3: N-terminal domain.

**Fig 3:**
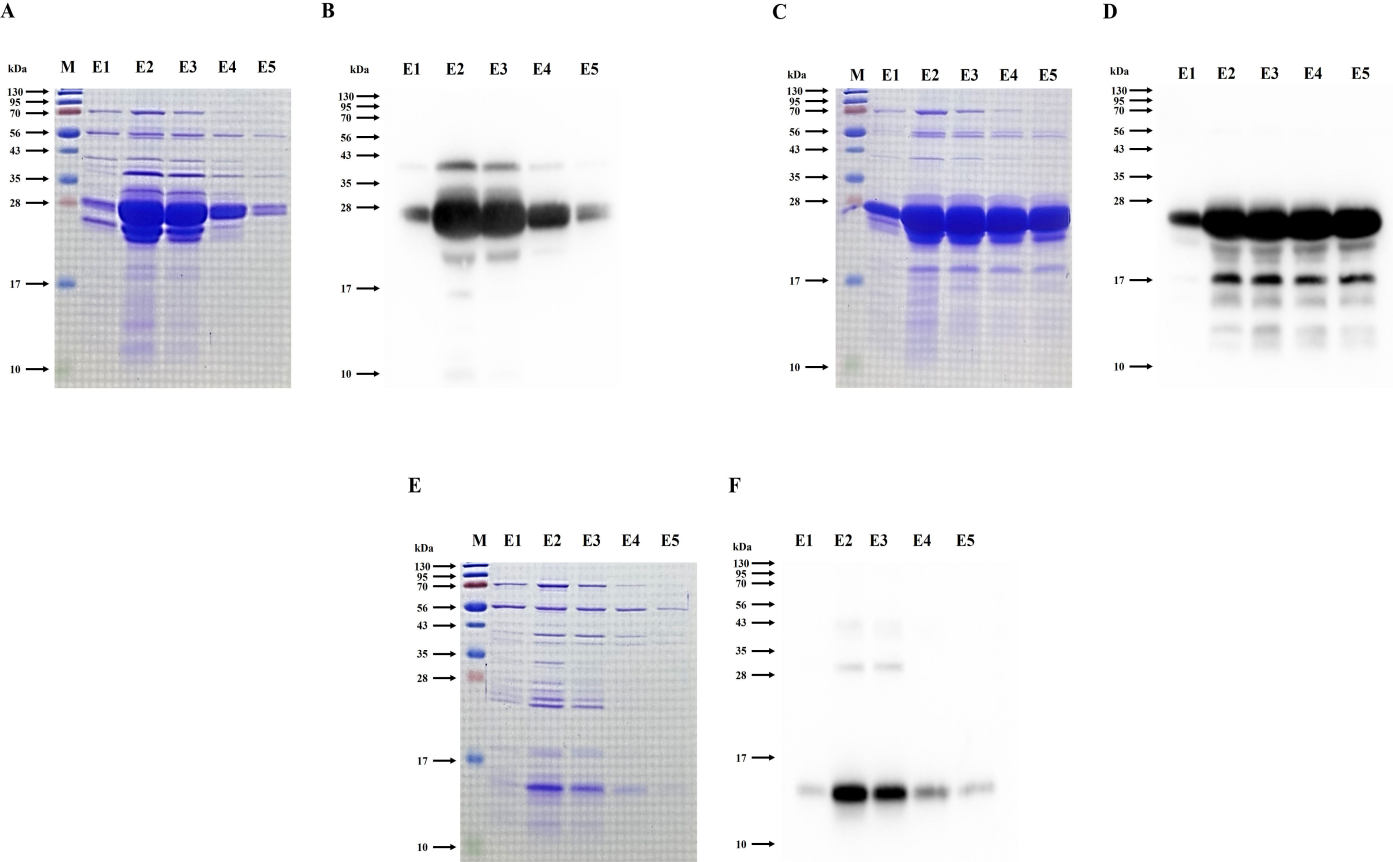
Purification of recombinant CAP10-3 endolysin and its domains using Ni-NTA affinity chromatography. **A, C, and E** show the SDS-PAGE analysis of purified FL endolysin, N-terminal domain, and C-terminal domain, respectively, while **B, D, and F** represent Western blot analysis in the same order. M: protein marker, E1-E5. Elution samples of respective proteins.

Protein expression for FL (∼35 kDa), N-terminal domain (∼27 kDa), and C-terminal (∼15 kDa) was confirmed through SDS-PAGE and Western blot analysis (Fig. 2A and B). Recombinant proteins were purified from the lysate supernatant using an open column with 2 mL of Ni-NTA slurry. After purification, the presence of recombinant endolysin and its domains in the eluted samples was confirmed via SDS-PAGE and Western blot. Distinct bands corresponding to approximately 28 kDa (FL), 27 kDa (N-domain), and 15 kDa (C-domain) were observed for the purified eluted proteins in SDS-PAGE (Fig. 3A, C and E) and Western blot (Fig. 3B, D and F) analysis.

### Evaluation of antibacterial activity of recombinant endolysins in cell lysates

**Fig 4:**
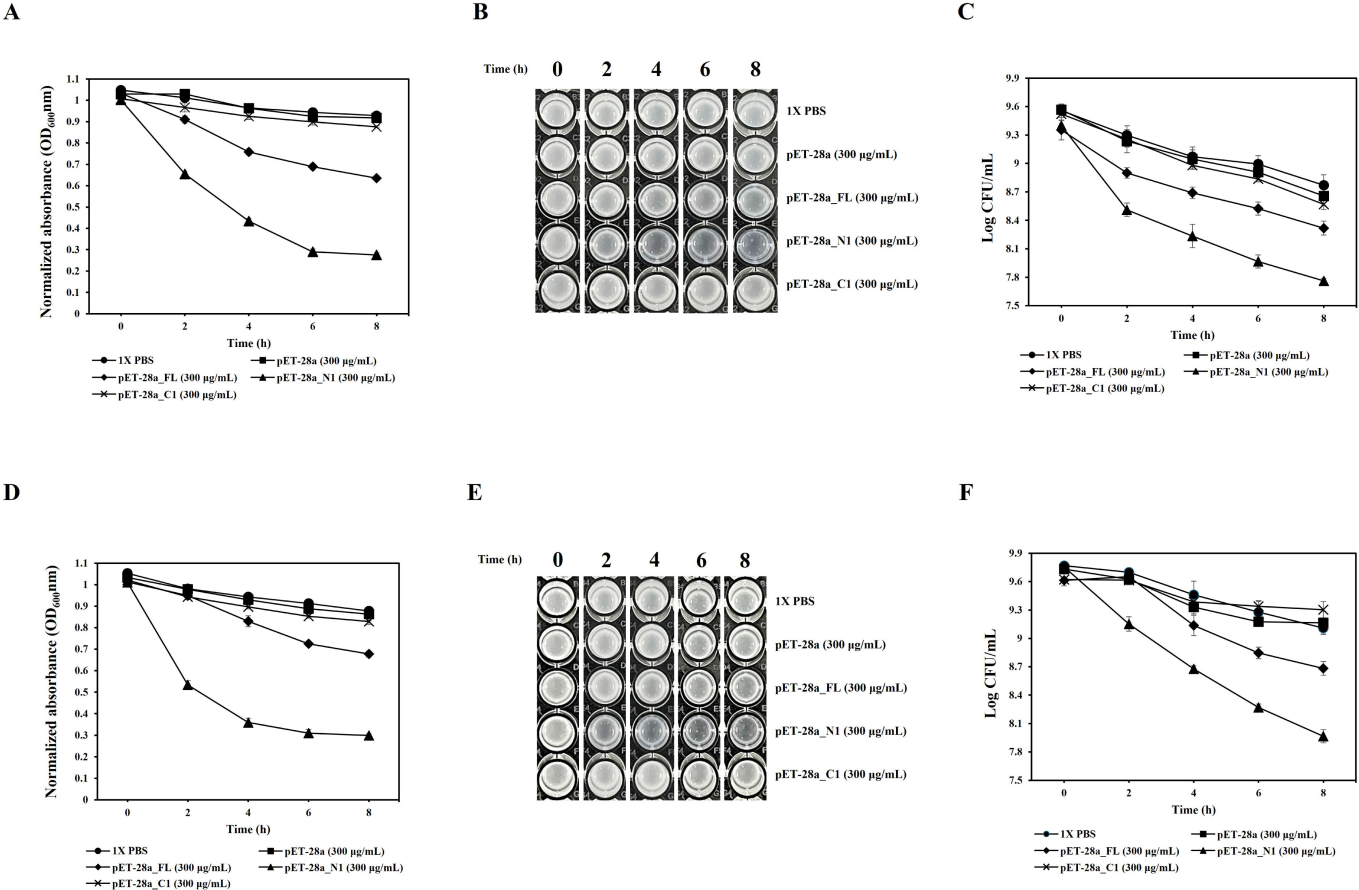
Antibacterial activity of recombinant endolysin and its domains in *E. coli* BL21(DE3) lysate supernatants over 8 h. **A and D** represent turbidity reduction through OD_600_**, B and E** represent visual assessment of bacterial turbidity, and **C and F** show viable cell counts of *C. acnes* KCTC 3314 and *C. acnes* KCTC 3320, respectively. FL (full-length endolysin), N1 (N-terminal domain), and C1 (C-terminal domain).

The lysate supernatants of pET-28α_CAP10-3 (FL) and pET-28α_CAP10-3_N1 (N-domain) significantly reduced turbidity from 2 h onward for both strains. At 8 h, the control pET28α lysate-supernatant maintained turbidity levels (OD_600_) of 0.917 and 0.863 for KCTC 3314 and KCTC 3320, respectively, like those of the 1X PBS control. In contrast, the pET-28α_CAP10-3 (FL) lysate supernatant reduced turbidity to 0.634 for KCTC 3314 and 0.677 for KCTC 3320. The pET-28α_CAP10-3_N1 (N-domain) lysate supernatant exhibited the greatest reduction, with turbidity levels of 0.275 for KCTC 3314 and 0.299 for KCTC 3320. Meanwhile, the pET28α_CAP10-3_C1 (C-domain) lysate supernatant resulted in turbidity reductions to 0.876 and 0.828 for KCTC 3314 and KCTC 3320, respectively, with significant reduction observed only for KCTC 3314 (*P* < 0.05) (Fig. 4A and D). Visual observations corroborated these findings, showing greater transparency in the lysates treated with pET-28α_CAP10-3 (FL) and pET-28α_CAP10-3_N1 (N-domain) (Fig. 4B and E). The viable cell count (Log CFU/mL) also correlated with the turbidity reduction results. The control pET-28α lysate supernatant caused minimal changes in viable counts after 8 h of incubation. The pET-28α_CAP10-3 (FL) lysate supernatant reduced the viable counts from initial 9.61 and 9.35 to 8.68 and 8.31 for KCTC 3314 and KCTC 3320, respectively. The pET-28α_CAP10-3_N1 (N-domain) lysate supernatant showed the most pronounced reduction, with viable counts dropping from 9.40 and 9.75 to 8.31 and 7.76 for KCTC 3314 and KCTC 3320, respectively. On the other hand, the pET-28α_CAP10-3_C1 (C-domain) lysate supernatant caused no significant reduction compared to the control, with values of 8.56 and 9.30 for KCTC 3314 and KCTC 3320, respectively (Fig. 4C and F) (*P* < 0.05).

### Evaluation of antibacterial activity of purified recombinant endolysins

**Fig 5:**
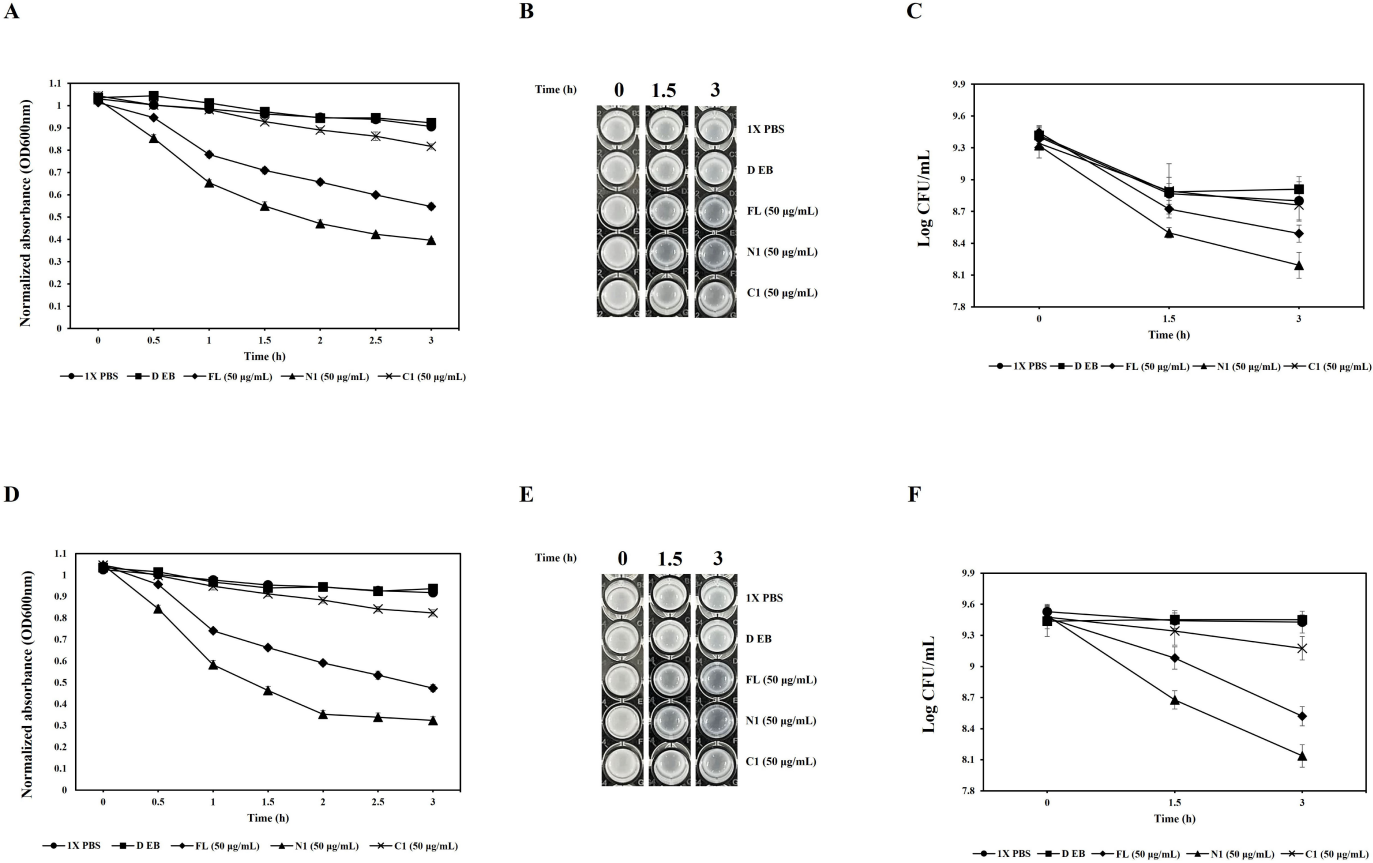
Antibacterial activity of purified recombinant endolysin and its domains over 3 h treatment. **A and D** represent turbidity reduction through OD_600_**, B and E** represent visual assessment of bacterial turbidity, and **C and F** show viable cell counts of *C. acnes* KCTC 3314 and *C. acnes* KCTC 3320, respectively. FL (full-length endolysin), N1 (N-terminal domain), C1 (C-terminal domain), and DEB (dialysis elution buffer).

Antibacterial activity of purified endolysin and its domains against both the *C. acnes* strains was evaluated using turbidity reduction assay where the controls (1× PBS and dialysis elution buffer [DEB]) had no significant impact on the turbidity (OD_600_ ≈ 1.0) of *C. acnes* KCTC 3314 and KCTC 3320. All purified recombinant endolysins significantly reduced turbidity by 3 h, with FL (50 µg/mL) and N1 (N-domain, 50 µg/mL) showing significant reductions starting at 0.5 h. By 3 h, FL reduced turbidity to 0.547 and 0.474 for KCTC 3314 and KCTC 3320, respectively, while N1 achieved the most substantial reductions to 0.395 and 0.324 for KCTC 3314 and KCTC 3320, respectively. C1 (C-domain, 50 µg/mL) reduced turbidity to 0.816 and 0.824 for KCTC 3314 and KCTC 3320, respectively, with significant reductions observed only for KCTC 3314 (*P* < 0.05) (Fig. 5A and D). Visual examination also revealed greater transparency in FL- and N1-treated samples compared to the controls and C1 (Fig. 5B and E). The viable cell count results replicated the turbidity data. DEB-treated samples showed no significant reduction in viable counts. In contrast, FL-treated samples reduced viable counts from 9.44 and 9.46 to 8.49 and 8.52 for KCTC 3314 and KCTC 3320, respectively. N1-treated samples exhibited the most significant reductions, with viable counts decreasing from 9.32 and 9.48 to 8.19 and 8.13 for KCTC 3314 and KCTC 3320, respectively. C1-treated samples showed no significant reduction for KCTC 3314, with counts of 8.76 compared to 9.34 initially, but significantly reduced counts for KCTC 3320, from 9.47 to 9.17 (Fig. 5C and F) (*P* < 0.05).

**Fig 6:**
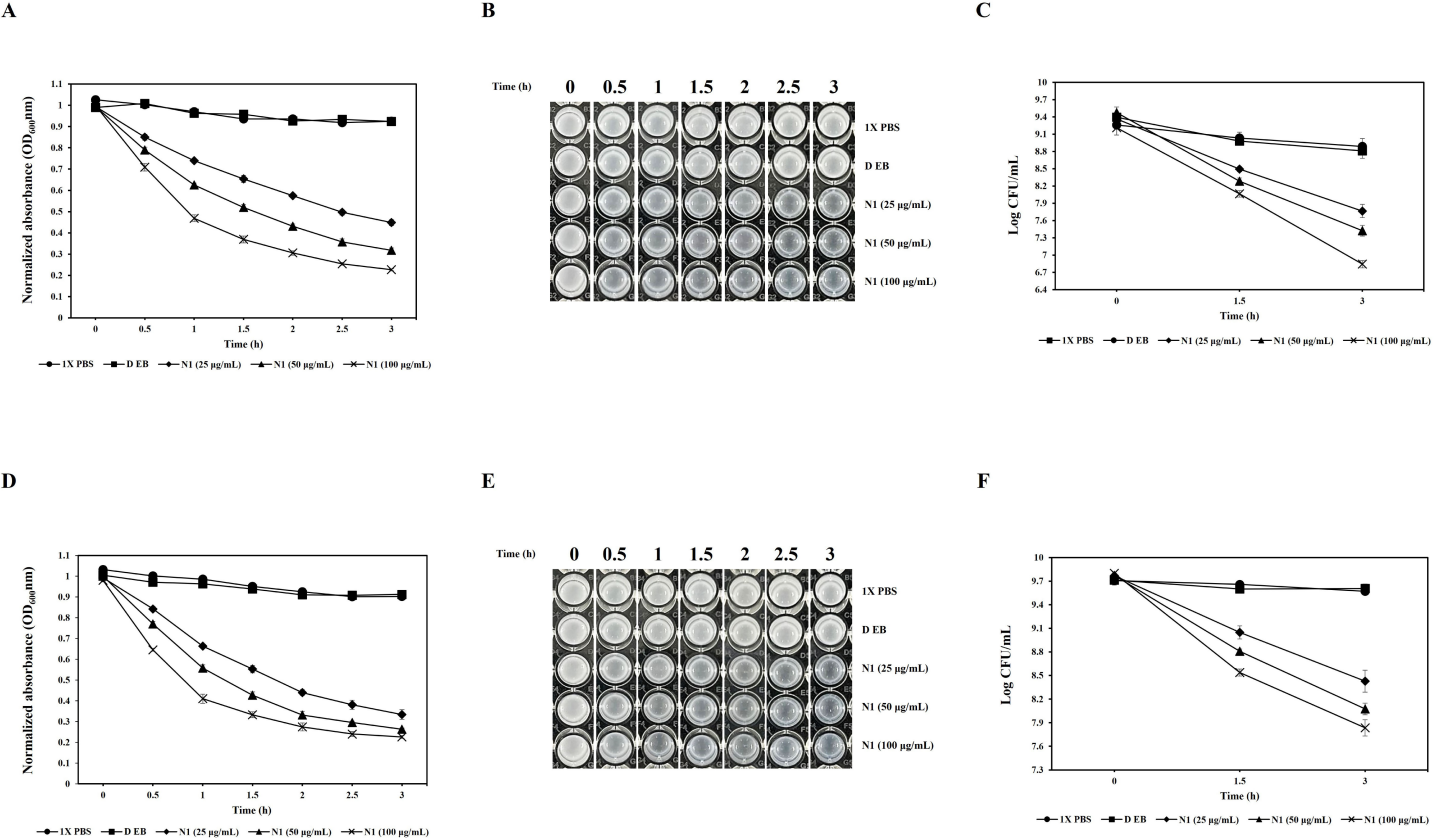
Dose-dependent antibacterial activity of the N-terminal domain against *C. acnes* KCTC 3314 and KCTC 3320. **A and D** represent turbidity reduction through OD_600_**, B and E** represent visual assessment of bacterial turbidity, and **C and F** show viable cell counts of *C. acnes* KCTC 3314 and *C. acnes* KCTC 3320, respectively. N1 (N-terminal domain), DEB (dialysis elution buffer)

Since the N-terminal domain displayed the highest antibacterial activity against both the strains, it was further analyzed for its anticipated dose-dependent effect. In the turbidity reduction assay, the control group (DEB) did not significantly affect the 0-h turbidity (OD_600_ ≈1.0) of *C. acnes* KCTC 3314 and KCTC 3320. While the N-domain exhibited a reduction in turbidity in all treatment groups (25, 50, and 100 µg/mL) starting from 0.5 h. After 1.5 h of incubation, the turbidity of KCTC 3314 decreased to 0.654, 0.520, and 0.369, respectively, and at the final 3 h of incubation, it decreased further to 0.448, 0.317, and 0.226, respectively. Similarly, at 1.5 h of incubation, the turbidity of KCTC 3320 decreased to 0.552, 0.427, and 0.333, respectively, and at the final 3 h of incubation, it decreased to 0.334, 0.262, and 0.226, respectively. This indicates that CAP10-3_N1 (N-domain) inhibits *C. acnes* KCTC 3314 and KCTC 3320 in a concentration-dependent manner (Fig. 6A and D) (*P* < 0.05). When visually examined, the turbidity of both KCTC 3314 and KCTC 3320 in the N1 (N-domain) 100 µg/mL treatment group became transparent at 0.5 h, while the 25 and 50 µg/mL treatment groups also showed reductions in turbidity (Fig. 6B and E). The results of viable cell counts (Log CFU/mL) showed a trend like that of the turbidity analysis. In the treatment groups with CAP10-3_N1 (N-domain) at concentrations of 25, 50, and 100 µg/mL, the initial viable cell count (Log CFU/mL) of KCTC 3314 decreased from 9.36, 9.47, and 9.21 to 7.76, 7.42, and 6.84, respectively, showing a concentration-dependent reduction. The same trend was observed for KCTC 3320, where the initial viable cell count (Log CFU/mL) decreased from 9.77, 9.77, and 9.79 to 8.42, 8.07, and 7.83, respectively. Likewise, the viable cell counts of the treatment groups showed a concentration-dependent decrease at 1.5 h, and there were significant differences compared to the control group (DEB). A decreasing trend in viable cell counts due to concentration differences was also observed up to the final 3 h of incubation (Fig. 6C and F) (*P* < 0.05).

### Morphology analysis of *C. acnes* treated with N-domain

**Fig 7:**
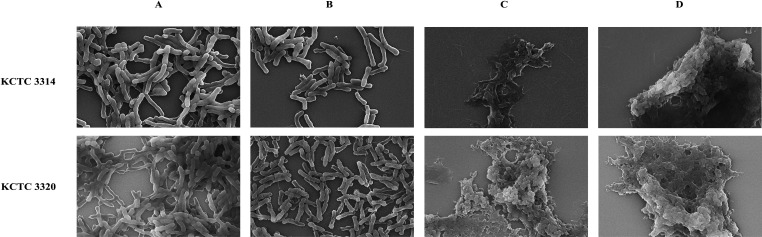
SEM analysis of *C. acnes* KCTC 3314 and KCTC 3320 after treatment with the N-domain of CAP10-3 endolysin. (**A, B**) Untreated and control-treated cells. (**C, D**) N-terminal domain-treated cells. The images highlight regions where cell lysis was evident, demonstrating the bacteriolytic activity of the endolysin. These fields were specifically selected to show the morphological changes associated with lysis and are not intended to represent the overall treated population.

The SEM images of *C. acnes* KCTC 3314 and *C. acnes* KCTC 3320 revealed that the untreated control (1× PBS) and the control (DEB) maintained intact cell surfaces with no visible damage (Fig. 7A and B). However, after 3-h treatment with CAP10-3_N1 (N-domain), rough cell surfaces and shriveled cell shapes were observed. Furthermore, the PG layer of the cells underwent lysis, causing the cells to burst and form sticky aggregates (Fig. 7C and D).

### Comparative inhibitory effects on skin commensal bacteria

**Fig 8:**
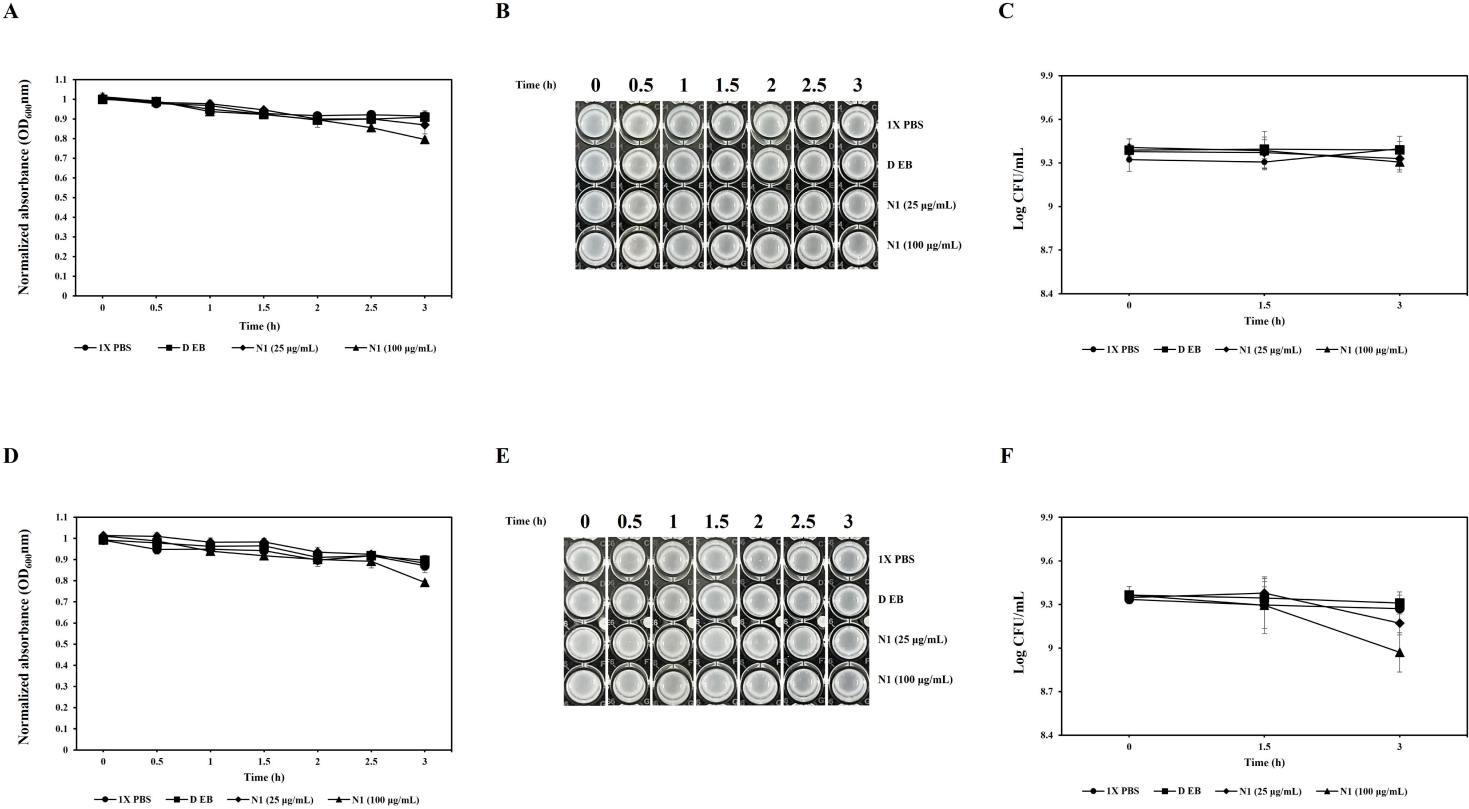
Antibacterial activity of the N-terminal domain against *S. aureus* KCTC 3881 and *S. epidermidis* CJNU 0702. **A and D** represent turbidity reduction through OD_600_**, B and E** represent visual assessment of bacterial turbidity, and **C and F** show viable cell counts of *S. aureus* KCTC 3881 and *S. epidermidis* CJNU 0702, respectively. N1 (N-terminal domain), DEB (dialysis elution buffer).

The results from the turbidity reduction assay showed that only higher concentrations of N-domain showed significant inhibitory effects on *S. aureus* KCTC 3881 and *S. epidermidis* CJNU 0702. N-domain, at 25 µg/mL, slightly reduced the turbidity to 0.869 and 0.883 for *S. aureus* KCTC 3881 and *S. epidermidis* CJNU 0702, respectively, after 3 h. However, N-domain, at 100 µg/mL, significantly reduced the turbidity to 0.795 and 0.791 for *S. aureus* KCTC 3881 and *S. epidermidis* CJNU 0702, respectively, after 3 h (Fig. 8A and D) (*P* < 0.05). When visually inspecting turbidity, it was difficult to distinguish the effects of 25 and 100 µg/mL of N1 (N-domain) as compared to the negative controls (Fig. 8B and E). Results of viable cell count (Log CFU/mL) showed that the negative controls didn’t show any notable changes in cell count for both *S. aureus* KCTC 3881 and *S. epidermidis* CJNU 0702. Treatment with 25 µg/mL of N1 (N-domain) resulted in a slight reduction in viable cell counts, with *S. aureus* KCTC 3881 decreasing from 9.37 to 9.32 Log CFU/mL and *S. epidermidis* CJNU 0702 decreasing from 9.34 to 9.17 Log CFU/mL. However, these reductions were not statistically significant compared to the control (DEB). On the other hand, treatment with 100 µg/mL of N1 (N-domain) reduced the viable counts of *S. aureus* KCTC 3881 from 9.40 to 9.30 Log CFU/mL, though this reduction was also not statistically significant. For *S. epidermidis* CJNU 0702, treatment with 100 µg/mL of N1 (N-domain) significantly reduced the viable count from 9.36 to 8.97 Log CFU/mL compared to the control (DEB) (Fig. 8C and F) (*P* < 0.05).

## DISCUSSION

The role of *C. acnes* in a variety of inflammatory and infectious disorders is becoming more rapidly acknowledged. In addition to its well-established involvement in acne vulgaris, its role in implant-associated problems such as infections linked to orthopedic devices and prosthetic joints has been reported as well. Furthermore, *C. acnes* has been linked to immune-mediated diseases like sarcoidosis and autoinflammatory illnesses like SAPHO syndrome (synovitis, acne, pustulosis, hyperostosis, osteitis), underscoring its wider pathogenic potential (30). Like other bacterial infections, disorders associated with *C. acnes* are primarily managed through antibiotic therapy. However, the increasing emergence of antibiotic-resistant strains, both against topical and systemic antibiotics (31, 32), poses significant challenges to current treatment strategies, emphasizing the need for additional effective therapeutic alternatives.

Bacteriophage therapy presents distinct advantages over conventional antibiotic treatments, including specificity to bacterial host, minimal-to-no harm to the human host or the commensal bacterial species, self-replication, and activity against multidrug-resistant strains. Furthermore, the genetic engineering of bacteriophages allows for enhanced antibacterial properties, such as the disruption of biofilms. Despite these advantages, challenges like the potential for bacterial resistance to phages, difficulties in the formulation and stabilization of phage preparations, host immune responses, and the risk of horizontal gene transfer of antibiotic resistance genes remain significant obstacles. Consequently, bacteriophage therapy has yet to be established as a mainstream therapeutic approach (33). Bacteriophage-derived lysins, or endolysins, are promising alternatives to antibiotics and phages due to their rapid bactericidal activity and low resistance potential. Unlike antibiotics, endolysins act within seconds by degrading the PG layer, and they target conserved cell wall structures critical for bacterial survival. They can effectively penetrate and disrupt biofilms, with some, like CF-301, eradicating them within hours. Endolysins also exhibit synergy with antibiotics, enhancing bacterial clearance and restoring antibiotic sensitivity. Unlike phages, they have predictable pharmacokinetics, and despite being immunogenic, their activity remains unaffected by neutralizing antibodies, supporting repeated use (34).

In this study, structural prediction of recombinant endolysin using AlphaFold revealed that its C-terminal domain is mainly composed of α-helices, while the N-terminal domain contains a combination of α-helix and β-sheets. Mayer et al. (35) also reported that the catalytic domain of endolysin CD27L consists of α/β fold. Similarly, α-helices seem to be the common secondary structure adopted by the endolysins derived from *C. acnes* phages (Fig. S1D). Endolysin from CAP 10-3 exhibited high sequence identity (92.28%) with *Corynebacterium kroppenstedtii* N-acetylmuramoyl-L-alanine amidase (A0A2W5SMS7.1. A), with an average model confidence (pLDDT) of 91.12. N-acetylmuramoyl-L-alanine amidase (EC 3.5.1.28) is a hydrolase found in human serum and various bacterial species, known to hydrolyze the amide bond between N-acetylmuramic acid and L-alanine in the bacterial cell wall (36–38). Thus, the endolysin from CAP 10-3 belongs to the amidase group of endolysins targeting amid bond in the PG of target bacterium. Similar predictions about the endolysins from phages infecting *C. acnes* have been made previously (29).

Previous study confirmed the genomic characteristics and antimicrobial activity of the CAP10-3 derived recombinant endolysin (27). However, low protein yield was observed (unpublished data), and further characterization of endolysin’s individual domains was not performed. In the current study, the expression yield (in soluble form) was significantly improved using the pET-28α expression vector which resulted in the production of soluble functional protein. To determine the functional aspects of C and N-terminal domains with respect to their antimicrobial activity potential, recombinant endolysin was expressed separately as FL, N-domain, and C-domain. Identifying the EAD and CBD in endolysins through domain segmentation has been extensively explored in other phages’ endolysins (10, 39).

Upon IPTG induction, the observed molecular size of each domain was larger than expected, likely due to the presence of His-tags at both the N- and C-domains in the pET-28α expression vector, as previously reported (40). Additionally, during purification, the FL endolysin’s band appeared smaller in size as compared to the band in the cell pellet, suggesting that the C-domain might have undergone proteolytic degradation, structural instability due to hydrophilic residues, or denaturation and degradation under specific conditions (pH, temperature, and ionic strength) (41, 42). To confirm C-domain degradation, mass spectrometry analysis is planned.

Genomic studies of *C. acnes* bacteriophages have predicted that their endolysins typically contain an amidase-type EAD in the N-domain, with the C-domain likely serving as a CBD and not involved in activity (29). Consistent with these findings, the endolysin from *C. acnes* phage CAP10-3 demonstrated effective lysis of *C. acnes* KCTC 3314 and KCTC 3320 by targeting their PG layer. The N-terminal domain of the recombinant endolysin not only maintained its activity but also exhibited higher antibacterial activity as compared to FL or the C-domain against both the tested *C. acnes* strains. Various other studies have reported varying results about the activity of truncated endolysins. While some found that the presence of CBD is an absolute requirement for endolysin activity and the CBD deletion/mutation results in the loss of activity (43, 44), other studies indicate that truncated endolysins are not only active but also more efficient than the FL endolysins (45, 46). The conflicting observations likely stem from structural variability, differences in the target bacterium PG, and the role of CBD in substrate recognition and binding. Moreover, the observed differences in activity between various FL endolysins and their N-terminal catalytic domains may also originate from how the CBD domain influences substrate access and enzyme efficiency. In some endolysins, like CD27L, the catalytic domain alone is more active because the binding domain mainly localizes the enzyme to the cell wall but may hinder catalytic activity. In some endolysins, on the other hand, the binding domain increases activity by facilitating the catalytic domain’s access to or tethering to the substrate, with both domains working synergistically. These variations reflect evolutionary adaptations to act from inside bacterial cells and suggest that removing the binding domain might improve therapeutic lysins by increasing activity while preserving specificity. Further investigation into how the catalytic domain interacts with the bacterial cell wall and the role of the CBD in modulating activity and host specificity can enable the design of optimized endolysins (35). Understanding these relationships may help engineer enzymes with enhanced effectiveness improving their antibacterial potential while maintaining targeted action.

In further experiments, the purified N-terminal domain exhibited antibacterial activity in a dose-dependent manner, that is, the higher the concentration of the protein, the greater was its lytic effect. Varotsou et al. (28) also reported a dose-dependent effect of recombinant endolysin from a *C. acnes* phage. Moreover, several other endolysins targeting various bacterial hosts are known to have a dose/concentration and time-dependent cell lysis effect (35, 47, 48). Cell morphology analysis of the bacterial cells treated with N-terminal domain through SEM analysis showed clear lysis of the target cells further confirming the lytic activity of the endolysin being studied.

Furthermore, the CAP10-3-derived endolysin showed minimal inhibitory effects on *S. aureus* KCTC 3881 and *S. epidermidis* CJNU 0702, which are representative commensal skin bacteria. Thus, the results of this study suggest that this endolysin is highly specific to *C. acnes*, and it can be successfully used to target *C. acnes* specifically. An endolysin killing *Enterococcus faecalis* also exhibited similar type of specificity, where it didn’t affect other bacterial species from the same (*Enterococcus*) genus (49). Most endolysins indicate near-species specificity, which is considered one of their most promising features. It helps in not only minimizing the adverse effects on commensals but also aids in minimizing the emergence of broad-range resistance, avoiding selective pressure on unintended bacterial species (50).

C-terminal domain alone exhibited minimal antimicrobial activity, potentially acting through a mechanism similar to antimicrobial peptides. Cationic α-helical peptides penetrate the porous PG layer of gram-positive bacteria and interact with the negatively charged lipid membrane, increasing membrane permeability or forming pores that lead to bacterial cell death (51, 52). The C-domain of CAP10-3, which is predicted to have an α-helical structure, may have exhibited antimicrobial activity by penetrating PG pores and disrupting membrane integrity under the experimental pH conditions. Thus, the C-domain’s main function is likely to act as a CBD, which warrants further investigation. Future studies incorporating fluorescent (green fluorescent protein) tagging could confirm its binding affinity to *C. acnes* PG (10, 53).

While recombinant endolysins have been extensively studied against pathogenic bacteria such as *S. aureus* and *Clostridium* spp. (39, 54–59), research on *C. acnes*-specific recombinant endolysins remains limited. This study highlights the potential of CAP 10-3-derived recombinant endolysin, especially its N-terminal domain, as a novel protein-based therapy for *C. acnes*, paving the way for innovative treatments in dermatology.

## Data Availability

No data set was generated in the current study.

## SUPPLEMENTAL MATERIAL

Fig. S1 aem.01168-25-s0001.docx Predicted secondary structures of selected endolysins.

ASM does not own the copyrights to Supplemental Material that may be linked to, or accessed through, an article. The authors have granted ASM a non-exclusive, world-wide license to publish the Supplemental Material files. Please contact the corresponding author directly for reuse.
