# Supplementary material for: Comparative antibacterial activity of N-terminal and C-terminal domains of a recombinant endolysin against Cutibacterium acnes
Source: Appl Environ Microbiol. 2025 Sep 22;91(10):e01168-25. doi: 10.1128/aem.01168-25 (PMC12542643; doi:10.1128/aem.01168-25)
Supplement: Fig. S1 — Predicted secondary structures of selected endolysins. [file aem.01168-25-s0001.docx]

**Predicted secondary structures of C- and N-terminal domains of endolysins from *C. acnes* phages (**using Alpha Fold**) along with their respective NCBI reference IDs**


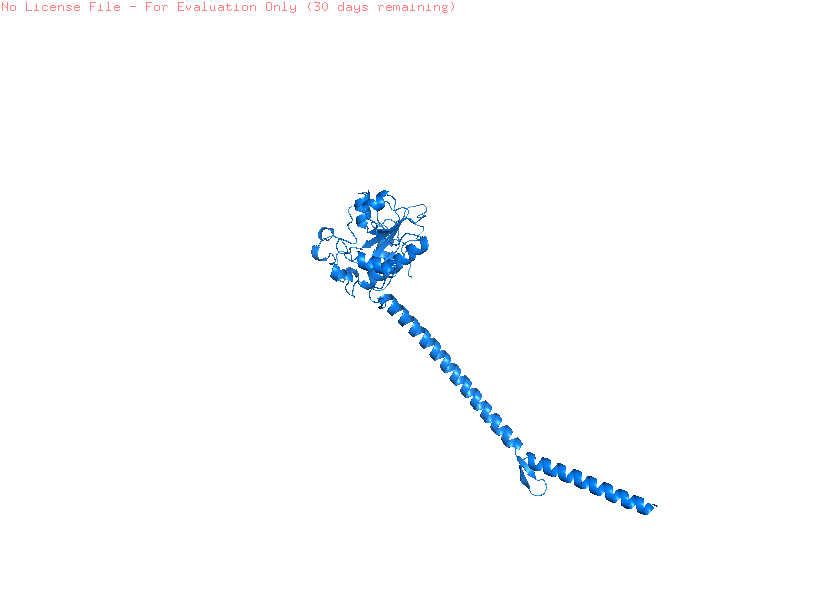


N-terminal domain

C-terminal domain

**Fig. S1A** *Propionibacterium* phage ATCC29399B_C **(**Accession ID# JX262225.1)


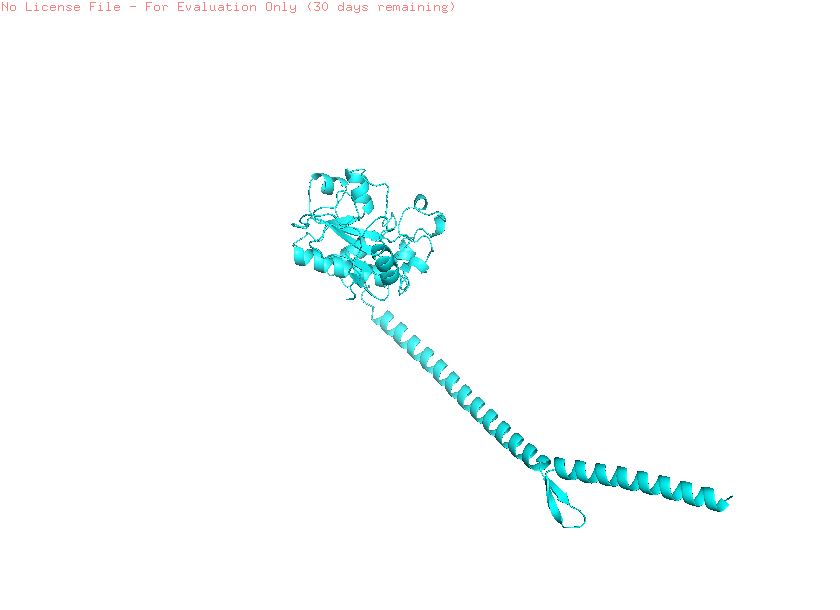


N-terminal domain

C-terminal domain

**Fig. S1B** *Propionibacterium* phage Pirate (Accession ID# NC_027623.2)


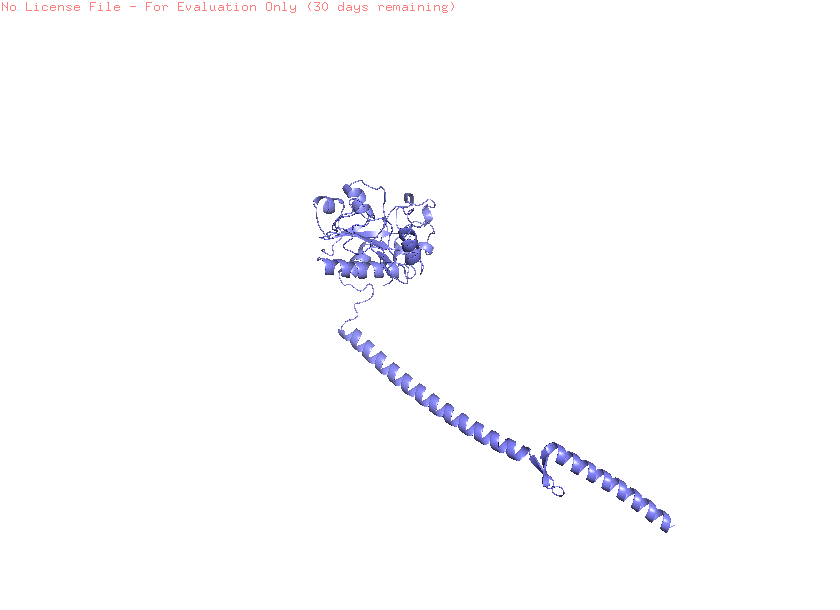


N-terminal domain

C-terminal domain

**Fig. S1C** *Propionibacterium* phage Stormborn (Accession ID# NC_027622.1)


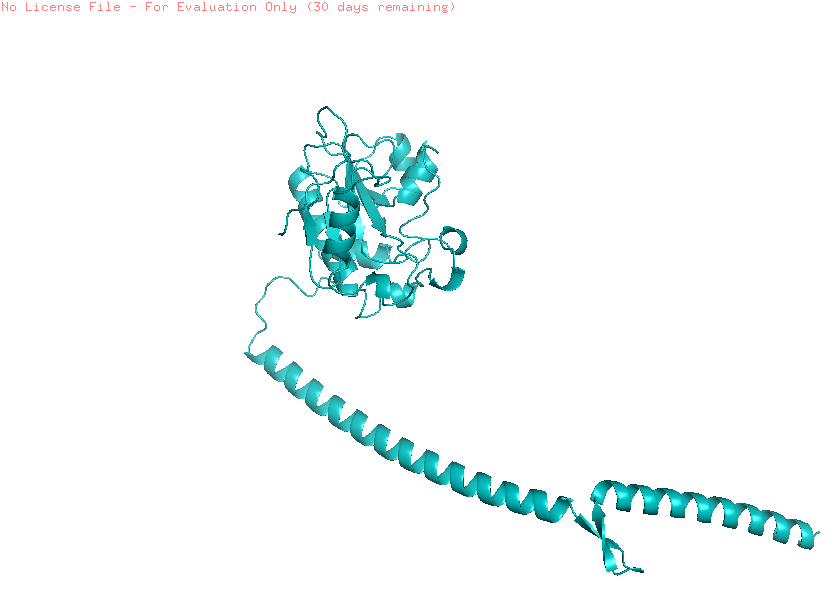


**Fig. S1D** *Propionibacterium* phage MrAK **(**Accession ID# NC_027620.1)

N-terminal domain

C-terminal domain
